# A case of urinary calculus in a patient with Ehlers‐Danlos syndrome

**DOI:** 10.1002/iju5.12587

**Published:** 2023-04-03

**Authors:** Shunsuke Owa, Yuna Hattori, Shigenori Yonemura, Masaki Sakurai

**Affiliations:** ^1^ Department of Urology Matsusaka Municipal Hospital Matsusaka Japan

**Keywords:** Ehlers‐Danlos syndrome, transurethral lithotripsy, ureteral calculus

## Abstract

**Introduction:**

Treatment of urinary tract calculi in patients with Ehlers‐Danlos syndrome, a connective tissue disorder, has rarely been reported.

**Case presentation:**

A 33‐year‐old woman with Ehlers‐Danlos syndrome sought evaluation of right‐sided abdominal pain from her family physician. Right‐sided hydronephrosis was noted and she was referred to our hospital for further evaluation and treatment. A ureteral calculus with a maximum diameter of 8 mm was demonstrated at the right ureterovesical junction. Transurethral lithotripsy was performed under general anesthesia without complications.

**Conclusion:**

Lithotripsy may be safely performed in patients with Ehlers‐Danlos syndrome.

**Keynote message**

Transurethral lithotripsy may be safely performed in patients with Ehlers‐Danlos syndrome.

**Abbreviations & Acronyms**
CT: computed tomography
EDS: Ehlers‐Danlos syndrome
ESWL: Extracorporeal shock wave lithotripsy
HU: Hounsfield unit
TUL: transurethral lithotripsy

## Introduction

EDS is a rare disorder in which connective tissues are affected. We describe a patient with EDS who underwent TUL for a ureteral calculus.

## Case report

A 33‐year‐old woman sought evaluation of right‐sided abdominal pain. She had EDS and was bedridden. Her hip joint was contracted and she had severe scoliosis, for which a spinal fusion was performed. Her right lung was atrophied due to scoliosis, and a tracheotomy was performed due to repeated pneumothoraces; she was placed on a ventilator at night. She had no intellectual impairment. Abdominal ultrasonography and CT were performed, revealing a right ureteral calculus in the right distal ureter (maximum diameter, 8 mm) and right hydronephrosis.

**Fig. 1:**
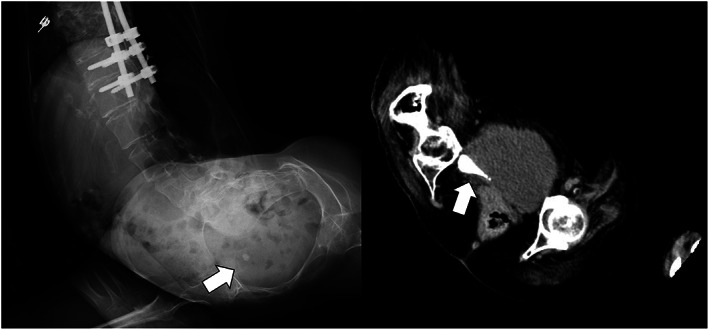
Imaging test results prior to treatment. A calculus with a maximum diameter of 8 mm was found in the lower ureter, and a calcified reservoir was found in the bladder.

Accumulation of high CT value material was also detected in the bladder (Fig. 1). The maximum CT value for the ureteral calculus was 1473 HU. ESWL was first considered as a minimally invasive treatment; however, the patient had severe joint contractures and difficulty breathing when supine on the lithotripter, making it difficult to perform the procedure. ESWL under ventilator management was considered, but returning to the hospital was difficult when multiple crushes were required, due to poor activities of daily living. Therefore, TUL was performed to crush the ureteral calculus.

The lower extremity contractures did not affect the TUL. When the cystoscope was inserted, large amounts of sandy calculi were noted in the bladder and removed. We were cautious when approaching the ureter to avoid mucosal injuries due to physical dilation of the ureter. We used the smallest diameter ureteral fiber we could prepare, a WiScope single‐use digital flexible ureteroscope® (8.6 Fr; OTU Medical, Union City, CA, USA). A ureteral access sheath was not used because of the increased risk of damaging the ureteral mucosa. The perfusate was pressurized with a Heiwa PC2® (Heiwa Medical Instruments, Co., Yamaguchi, Japan). The pressure setting for pressurization was changed from 200 mmHg (normal setting) to 100 mmHg to prevent excessive mucous membrane development due to water pressure. We did not measure the internal pressure in the urinary tract.

**Fig. 2:**
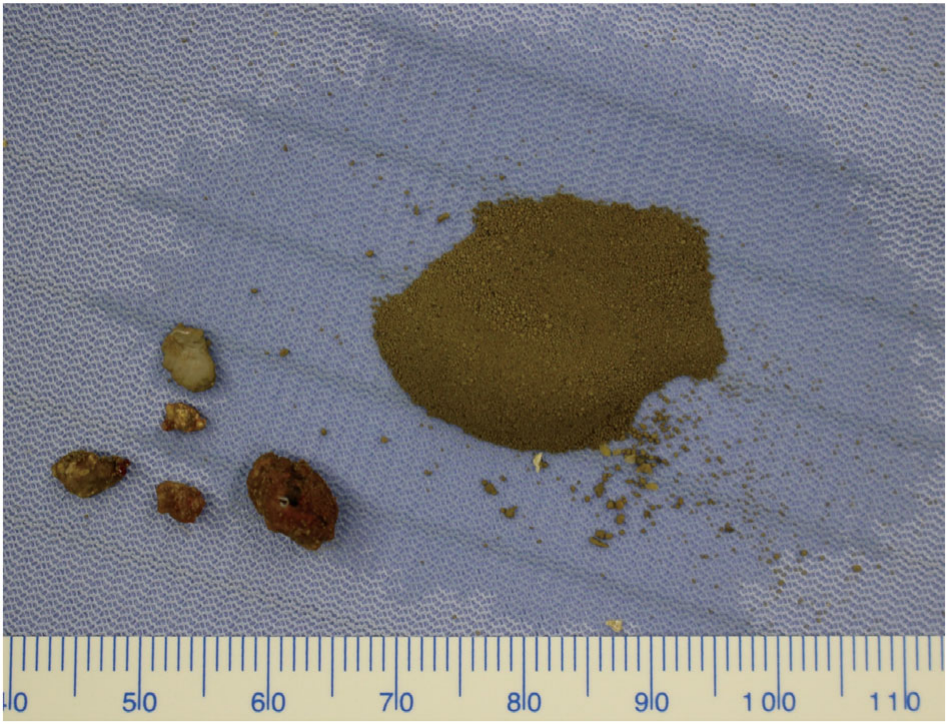
Calculi removed from the patient's body by treatment. The coarse calculus fragments on the left were present in the urinary tract, and the sandy calculi on the right were stored in the bladder.

**Fig. 3:**
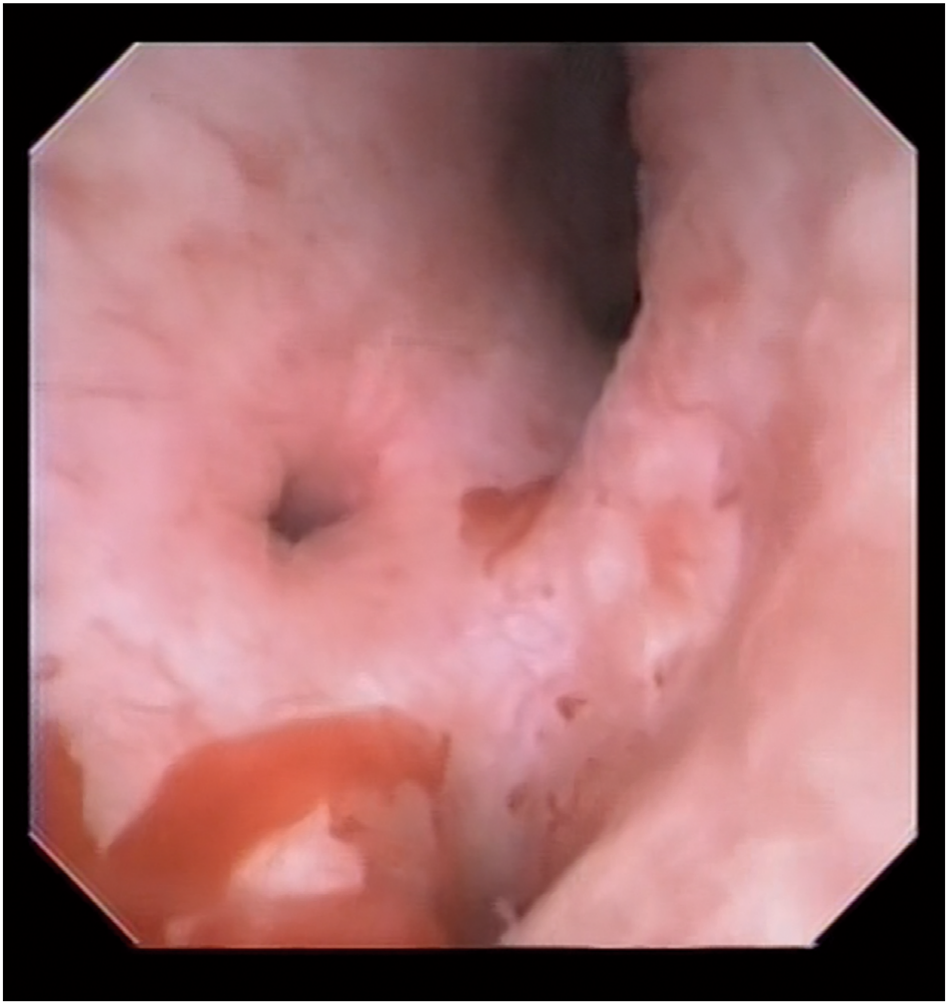
Image of the renal pelvis mucosa after calculus crushing. The reflux fluid was low pressure and the renal pelvis had minimal dilation, but bleeding from the mucosa was observed. There was no evidence of malignancy or inflammation, and the bleeding was thought to be due to slight mucosal dilation.

The ureteral fiber was inserted without difficulty. The ureteral calculus was immediately identified, crushed, and the fragments were removed (Fig. 2). The fiber was then advanced into the renal pelvis to ensure that there were no uncollected calculi. The mucosa within the urinary tract appeared normal to the unaided eye, but some mucosal bleeding was noted. There were no findings consistent with inflammation or other abnormalities at the bleeding site (Fig. 3). The entire procedure required 65 min. Minimal ureteral inflammation was noted intraoperatively and there was no mucosal damage to the ureteral orifice following intraureteral manipulation. Given the possibility of mucosal abrasion damage, ureteral stent placement after lithotripsy was not performed. The ureteral calculus was equally comprised of calcium oxalate and calcium phosphate. The sandy bladder calculi stored in the bladder consisted of acidic ammonium urate, calcium oxalate, and calcium phosphate. There was no evidence of bacteriuria postoperatively; however, the post‐void residual was >200 mL. Treatment with oral medications was ineffective, so intermittent catheterization was continued.

## Discussion

EDS is a disease characterized by fragility of connective tissues, such as skin, joints, and blood vessels due to genetic mutations involving collagen. EDS is classified in 13 subtypes based on clinical symptoms and genetic testing.^1^ EDS is known to cause various complications in blood vessels, skin, and the gastrointestinal tract, but reports involving treatment for urinary tract calculi are limited. There are no reports of urinary tract calculi occurring more frequently in patients with EDS than the general population. It is thought that the cause of urinary tract calculi in our case is largely due to low fluid intake. This mechanism is particularly likely because one‐half of the calculus within the ureter consisted of calcium oxalate. It has also been reported that the volume of residual urine after voiding may be 100–200 mL in EDS patients.^2^ This finding is thought to be due to non‐neurogenic bladder sphincter dysfunction,^3^ associated with an increased risk of urinary tract infection. Sandy calculi stored in the bladder contain high levels of calcium phosphate and ammonium urate, the formation of which may have been accelerated by chronic bacteriuria.

Although treatment with ESWL is generally minimally invasive, complications from shock waves have been reported.^4^ Specifically, a case of significant intra‐abdominal bleeding occurred after ESWL for a patient with EDS.^5^ Additionally, the major diameter index, calculated based on maximum HU and maximum diameter of the calculus, was 13 257 in this case. As the HU was >12 000, the probability of requiring more than one ESWL treatment was anticipated to exceed 50%.^6^ Based on the risk of complications and multiple treatments, we opted for TUL.

Indeed, the patient ultimately underwent TUL. We considered several points during treatment. First, we used the thinnest diameter fiber possible and did not use a ureteral access sheath, primarily because of concern about damage from physical mucosal dilation by the ureteroscope. As a result, no damage to the ureteral mucosa or opening occurred. Second, considering the fragility of urinary tract mucosa, we minimized the perfusate and operated without injecting perfusate if the visual field was clear. Although the causal relationship between high pressure in the urinary tract and mucosal injury in EDS patients is unclear, we observed bleeding from the renal pelvis mucosa in this case. There was no inflammation of the renal pelvis mucosa, so bleeding from the inflamed mucosa was unlikely, and there were few calculus fragments in the renal pelvis, so calculus damage was unlikely. The ureteral fiber was bleeding when the renal pelvis was reached, so bleeding due to abrasion by the fiber was also unlikely. No abnormal blood vessels were noted in the mucosa. Based on these findings, we believe that the bleeding may have been caused by hydraulic dilation of the mucosa. Another benefit of a low‐pressure perfusate is the prevention of postoperative infections.^7^ Therefore, it was thought that we should devise a way to make the pressure even lower than the setpoint we used. Risk factors for postoperative urinary tract infections following TUL include operative time, preoperative pyuria, and preoperative CT findings.^8^, ^9^, ^10^ Patients with EDS are at high risk of developing urinary tract infections,^2^ and pyuria suggests a relatively high risk of postoperative infections following upper urinary tract procedures.

In this case, we were able to successfully treat ureteral calculi in a patient with EDS. We hope that this case will help establish safe treatment of upper urinary tract calculi in patients with EDS.

## Conclusion

When urinary tract calculi occur in EDS patients, TUL can be safely performed by selecting the instruments to be used and making intraoperative adjustments.

## Author Contributions

**Shunsuke Owa:** Conceptualization; data curation; writing – original draft; writing – review and editing. **Yuna Hattori:** Supervision. **Shigenori Yonemura:** Supervision. **Masaki Sakurai:** Supervision.

## Conflict of interest

The authors declare no conflicts of interest.

## Approval of the research protocol by an Institutional Reviewer Board

Not applicable.

## Informed consent

Patient consent was obtained.

## Registry and the Registration No. of the study/trial

Not applicable.
